# Production of Anisotropic NdFeB Permanent Magnets with In Situ Magnetic Particle Alignment Using Powder Extrusion

**DOI:** 10.3390/ma18153668

**Published:** 2025-08-04

**Authors:** Stefan Rathfelder, Stephan Schuschnigg, Christian Kukla, Clemens Holzer, Dieter Suess, Carlo Burkhardt

**Affiliations:** 1 Institute for Precious and Technology Metals, Pforzheim University, Tiefenbronner Str. 65, 75175 Pforzheim, Germany; stefan.rathfelder@stud.unileoben.ac.at; 2 Department of Polymer Engineering and Science, Institute of Polymer Processing, Montanuniversitaet Leoben, Otto Gloeckel-Straße 2, 8700 Leoben, Austria; stephan.schuschnigg@unileoben.ac.at (S.S.); clemens.holzer@unileoben.ac.at (C.H.); 3 Research and Innovation Service, Montanuniversitaet Leoben, Franz-Josef-Strasse 18, 8700 Leoben, Austria; 4 Faculty of Physics, University of Vienna, Boltzmanngasse 1, 1090 Vienna, Austria; dieter.suess@univie.ac.at

**Keywords:** powder extrusion molding (PEM), metal injection molding (MIM), anisotropic NdFeB permanent magnets, recycling of end-of-life (Eol)-magnets

## Abstract

This study investigates the sustainable production of NdFeB permanent magnets using powder extrusion molding (PEM) with in situ magnetic alignment, utilizing recycled powder from an end-of-life (Eol) wind turbine magnet obtained via hydrogen processing of magnetic scrap (HPMS). Finite Element Method (FEM) simulations were conducted to design and optimize alignment tool geometries and magnetic field parameters. A key challenge in the PEM process is achieving effective particle alignment while the continuous strand moves through the magnetic field during extrusion. To address this, extrusion experiments were performed using three different alignment tool geometries and varying magnetic field strengths to determine the optimal configuration for particle alignment. The experimental results demonstrate a high degree of alignment (*B_r_/J_s_* = 0.95), exceeding the values obtained with PEM without an external magnetic field (0.78). The study confirms that optimizing the alignment tool geometry and applying sufficiently strong magnetic fields during extrusion enable the production of anisotropic NdFeB permanent magnets without post-machining, providing a scalable route for permanent magnet recycling and manufacturing. Moreover, PEM with in situ magnetic particle alignment allows for the continuous fabrication of near-net-shape strands with customizable cross-sections, making it a scalable approach for permanent magnet recycling and industrial manufacturing.

## 1. Introduction

The NdFeB permanent magnet, invented in 1983, still has the highest energy yield of all magnets [1]. This is why these magnets can be found in many high-tech applications, especially in the fields of electromobility, wind turbines, robotics and many other electrical applications. Therefore, permanent magnets based on NdFeB have an absolute key role in the green and digital transformation in Europe. These examples show that the demand for NdFeB permanent magnets will increase enormously in the coming years. For example, 95% of all electric cars are powered by traction motors containing rare earth magnets. Demand for rare earth permanent magnets (REPM) in the European Union (EU) was around 5000 t/year in 2019 and is expected to increase to 70,000 t/year by 2030. Much of the REPM used in the EU is imported from a single supplier country, which accounted for approximately 93% of the 130,000 tons produced worldwide in 2019 [2,3]. One way to reduce this dependency is to recycle the magnets in circulation in Europe. The EU-funded SUSMAGPRO and REESilience projects aim to find and recycle these sources of raw materials, creating a more stable supply chain [4,5].

NdFeB permanent magnets can be categorized according to the way they are manufactured. Sintered NdFeB permanent magnets are produced by powder metallurgy. This means that the powder is aligned in a mold, pressed and sintered [6]. These magnets have the highest energy product compared to the other manufacturing processes. For polymer-bonded magnets, the powder is mixed with a polymer binder, which allows the magnet to be produced using a polymer-based manufacturing process, such as injection molding or extrusion. As the polymer remains in the part, these magnets have a lower energy product than sintered magnets. A combination of these two types of magnets is known as MIM magnets. MIM stands for metal injection molding and, unlike bonded magnets, the polymer binder is removed to create a metallic structure. Isotropic NdFeB permanent magnets produced from recycled material with MIM have been reported by Burkhardt et al. [7], while Bioud et al. [8] demonstrated the fabrication of isotropic NdFeB magnets using virgin material in their study. Furthermore, Hartwig et al. [9] successfully produced anisotropic NdFeB permanent magnets from virgin material using the MIM process. The PEM (Powder Extrusion Molding) [10,11] process differs from MIM in the way the parts are shaped. Instead of injecting the feedstock into a mold at high pressure as in MIM, PEM uses an extruder to continuously push the feedstock through a die. A detailed description of the manufacturing process of isotropic NdFeB permanent magnets with PEM from recycled powder was published in a previous article [12]. The MIM/PEM process consists of four steps:

1. Compounding;
2. Shaping;
3. Debinding;
4. Sintering.

The first step is to mix the powder and binder; the mixture of powder and binder is called the feedstock. The second step is molding, either by injection molding or extrusion, depending on the process. The green parts cannot be used directly due to their thermal and mechanical instability, so the binder is removed using a debinding process tailored to the binder. The final step is to sinter the debinded brown part in a vacuum or inert gas atmosphere. The result of these processes are parts that combine the advantages of sintered magnets, such as very good mechanical properties, with the shaping effort of bonded magnets.

Unlike isotropic permanent magnets, in which the magnetic particles have uniform magnetic properties regardless of their orientation, anisotropic permanent magnets have a preferred orientation of the magnetic particles along a particular axis, known as the easy axis. To achieve this orientation, an external magnetic field must be applied during the manufacturing process. The orientation of the magnetic particles is described by the degree of alignment.

There are several ways to determine the orientation of the magnetic particles in permanent magnets [13,14]. Techniques such as electron backscatter diffraction (EBSD) [14] and X-ray diffraction (XRD) [15] allow for detailed analysis of the crystallographic texture and alignment of individual grains. However, these methods are time consuming as they require careful sample preparation. In addition to structural analysis methods, the degree of alignment can also be estimated by magnetic measurements performed parallel to the easy and hard axes. These measurements offer an initial indication of the magnetic particle alignment quality and allow for monitoring of the alignment during the production of anisotropic permanent magnets. A commonly used approach is to determine the ratio of the remanent polarization (*B_r_*) to the saturation polarization (*J_s_*), as shown in Equation (1):

(1) $$cos\varphi =\frac{B_{r}}{J_{s}}.$$

There are two ways to measure magnetic properties. In a vibrating magnetometer (VSM), the sample is made to vibrate in a magnetic field. The vibrations change the magnetic flux through the coils, inducing an electrical voltage. The voltage is proportional to the magnetization of the sample. The voltage can then be used to output the magnetic properties of the sample. The measurement is carried out in an open magnetic circuit, creating an internal magnetic field *H*_(internal)_ in the sample, which acts against the applied field *H*_(applied)_. The magnetic field in the sample is then described by Equation (2). The demagnetization factor *N* is a dimensionless value that varies depending on the shape of the material. The magnetization *M* describes how strongly a material reacts to an applied magnetic field. This adjustment is called demagnetization correction [16].

(2) $$H_{\left(internal\right)}=H_{\left(applied\right)}-NM$$

When measuring with a hystograph or hysteresis graph, the measurement is made with a constant flux change and with coils. The magnet is clamped between two pole shoes with a known field direction, and a coil sensor is placed around the magnet to measure the magnetic flux. On the pole pieces, there are coil windings that generate the magnetic field. The voltages measured in the coil sensor are used to determine the magnetic flux density of the pole.

In addition to the orientation of the magnetic particles, the density of the sintered sample plays a role in good remanence *B_r_*. According to Kaneko et al. [17] the remanence *B_r_* increases linearly with density, with the highest values observed in magnets reaching a relative density of 97%. Another point that affects remanence *B_r_* is the amount of the Nd_2_Fe_14_B phase and Nd-rich phase. A higher proportion of Nd-rich phase helps achieve a better coercivity as it can eliminate defects at the grain boundary and lead to a better decoupling of the grains. A large amount of non-magnetic Nd-rich phase therefore has a negative effect on remanence *B_r_*.

In the production of anisotropic MIM magnets [9] or pressed and sintered magnets [6], particle alignment takes place under static conditions within a mold or die. In contrast, in continuous manufacturing processes such as powder extrusion molding (PEM) or material extrusion (MEX), the alignment must occur as the particle/binder melt flows through a magnetic field during extrusion. Magnetic particle alignment during continuous flow has so far only been described in the context of MEX-processes of polymer-bonded magnets. Sarkar et al. [18] demonstrated an in situ alignment process using 3D filament printing with an NdFeB feedstock and a nylon binder. In their approach, an external magnetic field is applied at the nozzle of the printer, inducing rotation of the magnetic particles and alignment along the field direction. The polymer matrix, in which the particles are embedded, becomes fluid under heat, enabling the particles to rotate. As the melt passes through the magnetic field, the particles align in the direction of the field. Sarkar’s study emphasizes that the torque generated by the magnetic field must overcome the counteracting torque caused by the binder’s viscosity. The experiments, conducted with a feedstock of 65 vol% NdFeB + SmFeN in Nylon 12, show that increasing the field strength improves both the remanence and the degree of alignment. A mathematical model developed by Sarkar et al. [19] describes the interaction between viscous forces and particle rotation under an external magnetic field.

Sonnleitner et al. [20] also investigated magnetic particle alignment during the FFF process. They found that stronger magnetic fields up to 200 mT resulted in a higher *B_r_/J_s_* ratio, with a maximum value of 0.94 observed for strontium hexaferrite in PA12 at 53 vol%. Compared to lower field strengths of 100 mT and 150 mT, which resulted in *B_r_/J_s_* ratios of about 0.87 and 0.91, respectively, the higher field improved the alignment of the particles. Gandha et al. [21] used an extrusion-based 3D printing process to fabricate polymer-bonded magnets from anisotropic NdFeB powder. Magnetic alignment was attempted in situ using two sintered NdFeB permanent magnets mounted on the nozzle, but the resulting field strength was insufficient, leading to only a slight increase in remanence. Improved alignment was achieved through a subsequent post-printing alignment step.

This article presents a novel process for producing sintered, anisotropic NdFeB permanent magnets using the Powder Extrusion Molding (PEM) technique. The method enables the continuous fabrication of near-net-shape strands with aligned magnetic particles. To facilitate magnetic alignment during extrusion, various alignment tool geometries were developed and tested under different magnetic field strengths. All experiments were conducted using recycled NdFeB material recovered from an end-of-life wind turbine magnet. In contrast to conventional powder metallurgy processes, where the magnetic powder is first compacted into blocks and then sintered, followed by machining into the desired shape, the PEM process enables direct shaping during the extrusion step. This eliminates time-consuming processing steps such as grinding or wire-cutting. The ability to directly extrude near-net-shape geometries such as arc-segmented, rectangular, or bread-shaped cross-sections offers advantages for series production. These geometries are widely used in electric drives, showing the potential of the PEM process for industrial magnet manufacturing.

## 2. Experimental

### 2.1. Materials

The starting material for these experiments was obtained from an end-of-life (Eol) wind turbine magnet, which was processed into powder using the environmentally friendly and energy-efficient hydrogen processing of magnetic scrap (HPMS) method [22,23]. In this process, the magnet was exposed to hydrogen at a constant pressure of 3 bar until the reaction was complete. During hydrogenation, the magnet decomposed into Nd_2_Fe_14_BH_x_ and NdH_~2.7_ phases. The hydrated powder exhibits lower anisotropy and is difficult to align in a magnetic field due to disrupted exchange coupling [24,25]. To restore the anisotropic properties necessary for effective particle alignment and to prevent excessive grain growth during sintering, the hydrogen was removed by controlled degassing. For this purpose, the HPMS powder was treated under vacuum at 600 °C for 2 h in a vacuum furnace. Following degassing, the HPMS powder was milled using a Retsch Nano 500 MM ball mill in three cycles of 10 min each at 35 Hz to achieve a suitable particle size for extrusion and alignment. The particle size distribution of the milled powder was measured using a Mastersizer 3000 particle size analyzer from Malvern Panalytical GmbH (Nuremberg, Germany).

The binder system used in this work was developed by Burkhardt et al. [7]. The feedstock, consisting of powder and a binder system, is prepared in multiple steps. The binder system comprises a main binder composed of waxes, a backbone binder (polyethylene, PE) and an additive. In the first step, the powder is coated with the main binder inside a glove box filled with argon. For this purpose, the main binder is dissolved in a solvent and mixed with the powder. The coated powder is then dried under vacuum and subsequently granulated. In the second step, the granulated powder is compounded with the backbone binder in an extruder. The compounding procedure followed the approach described in [12].

### 2.2. Production of Anisotropic NdFeB Permanent Magnets via Powder Extrusion Molding Under an External Magnetic Field

**Figure 1:**
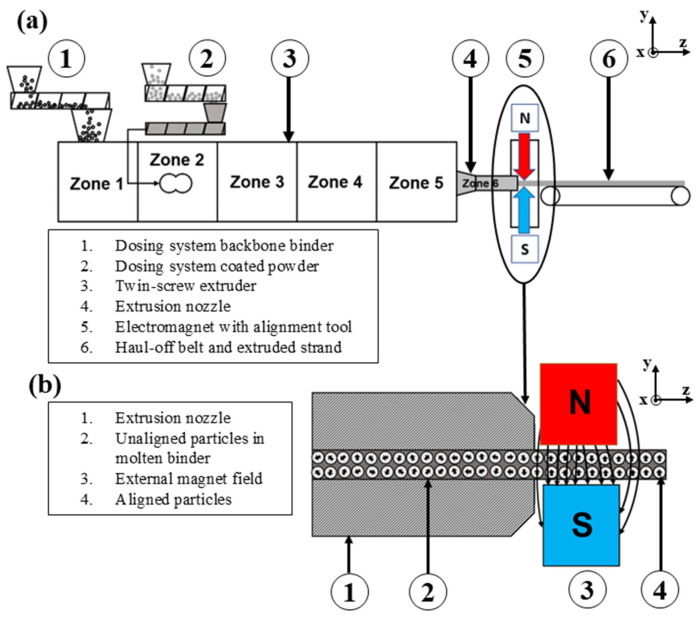
(**a**) Schematic of the powder extrusion molding (PEM) process with externally applied magnetic field, based on [12]. (**b**) Cross-sectional view of the extrusion nozzle, illustrating particle alignment in the molten binder under the influence of the external magnetic field.

Anisotropic NdFeB permanent magnets were produced using PEM and in situ particle alignment (Figure 1a). For these experiments, a KETSE 12/36 twin-screw extruder from Anton Paar TorqueTec GmbH (Duisburg, Germany) was used. The coated powder and the backbone binder are fed into the extruder via dosing systems (1) and (2). The magnetic field in this work is generated by an EM2000 electromagnet from Dr. Brockhaus Messtechnik GmbH & Co. KG (Lüdenscheid, Germany).

The coated powder and the backbone binder are mixed homogeneously using twin screws, while a nozzle (4) determines the cross-sectional shape of the strand. In this article, a bread loaf cross-section is extruded, a more detailed description is described in [12]. After shaping, the magnetic alignment of the magnetic particles takes place with an electromagnet (5), and a haul-off belt pulls off the strand (6).

The alignment process is shown in detail (Figure 1b). The extrusion die (1) forms the strand, but the magnetic particles are still unaligned (2). As the strand passes through the magnetic field (3), the magnetic particles align parallel to the applied field. When the strand leaves the magnetic field, the magnetic particles are aligned (4). The die is made of tool steel and therefore has a higher magnetic permeability than the molten feedstock being forced through the die. This causes the magnetic field lines to bypass the strand, and the magnetic particles are not going to be aligned. For this reason, alignment is carried out with the alignment tool after the strand has left the extrusion die but before it has solidified. According to Sarkar et al. [18], the degree of alignment during the extrusion of powder/binder mixtures depends on the strength of the magnetic field and the extrusion temperature, which necessitates thatthe binder remains in a molten state.

The extrusion parameters are adapted from previous work on producing isotropic NdFeB permanent magnets with PEM [12] are presented in Table 1. In this work, the focus is on determining a suitable magnetic field and a suitable tool geometry for alignment; therefore, all tests are carried out at the same extrusion temperature. The strands are produced with a powder filling of 50 vol%. The molded strands are aligned with three magnetic fields of differing strengths. The weakest magnetic field is induced with a 2 A current in the electromagnet, then the next strand is increased to 6 A, and the strongest magnetic field is created with a 10 A current in the electromagnet. This process allows for the ideal magnetic field strength to be determined. To be able to measure the magnetic values of the samples, rectangular samples measuring 5 mm × 5 mm × 3 mm are cut from the green part.

### 2.3. In Situ Alignment of Magnetic Particles with PEM

**Figure 2:**
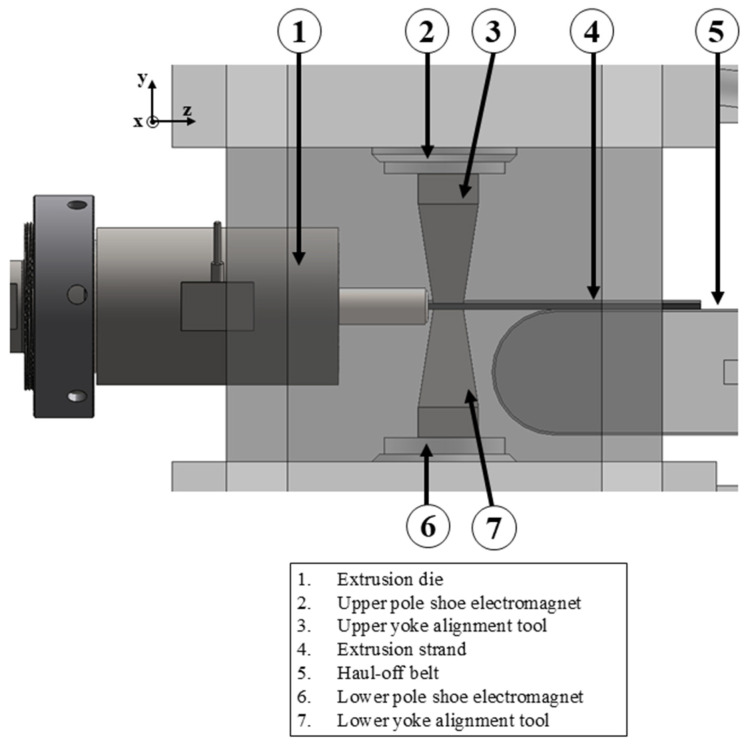
Alignment tool in detail.

The main objective of this work is to demonstrate that magnetic particles can be aligned in situ using the PEM process. This requires the development of a tool that directs the magnetic field generated by an electromagnet through the extruded strand. The maximum permissible current for the electromagnet is 10 A, although the exact number of windings and the winding diameter are not specified by the manufacturer. At higher currents, the electromagnet tends to overheat in continuous operation. Due to the current limit of 10 A, it is not possible to increase the magnetic flux density by increasing the current. It is therefore important to design a tool whose geometry and material properties generate an optimum magnetic field. However, this magnetic field must not be so strong that it stops or hinders the movement of the stranded wire as it passes through. A detailed 3D CAD model of the alignment tool installed in the electromagnet is presented (Figure 2).

The two yokes (3, 6) are mounted on the pole shoes (2, 7) of the electromagnet so that they transmit the magnetic flux generated by the electromagnet to the extrudate. Good transmission of the magnetic flux through the alignment tool leads to better alignment of the magnetic moments in the extrudate.

The permeability of a material exerts a significant influence on the efficacy of a tool in conducting magnetic flux density. Materials with high permeability facilitate the transmission of magnetic flux, exemplified by iron. Conversely, materials with low permeability impede the flow of magnetic flux, as observed in the case of air.

The permeability of materials is typically characterized in terms of relative permeability ${µ}_{r}$. This describes the relationship between the absolute permeability µ and the permeability of the vacuum ${µ}_{0}$, (Equation (3)):

(3) $${µ}_{r}=\frac{µ}{{µ}_{0}}.$$

In this work, the upper and lower yokes of the tool were made of steel S235JR. This is an unalloyed structural steel that is used for general engineering purposes. It is nevertheless ferromagnetic and can therefore conduct magnetic fields well. In addition to the permeability of a material, the shape of the tool can also exert an influence on the magnetic flux density. A reduction in the cross-sectional area results in an increase in the magnetic flux, which is then concentrated in a specific area.

The magnetic flux density *B* in Tesla [T] is calculated using the following equation:

(4) $$B=\frac{\Phi }{A}.$$

**Figure 3:**
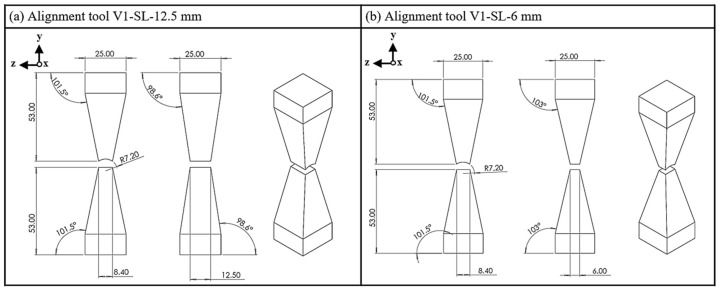
Alignment tool V1; (**a**) side length (SL), 12.5 mm; (**b**) side length, 6 mm.

In this equation, Φ denotes the magnetic flux (Wb), whilst *A* is representative of the cross-sectional area (m^2^). This article examines three tools that employ the principle of cross-sectional reduction to generate a maximum magnetic field and to align the magnetic particles in the strand parallel to this field. The first variant reduces the cross-sectional area through the tapered convergence of the upper and lower yokes (Figure 3). In the extrusion direction (z-direction), the examination focuses on a mold with dimensions of 12.5 mm in length and 6 mm in width. The tool has a width of 8.4 mm at the end of the cone, which corresponds to the width of the strand.

**Figure 4:**
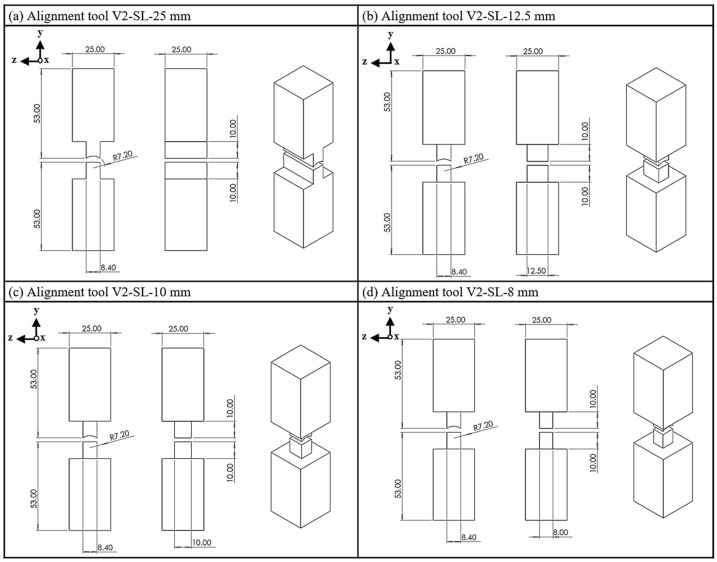
Alignment tool V2; (**a**) side length, 25 mm; (**b**) side length, 12.5 mm; (**c**) side length, 10 mm; (**d**) side length, 8 mm.

In the second geometry variant V2, there is no constant reduction in the cross-section, as in variant V1, but rather an abrupt reduction (Figure 4). Two steps are milled in the width of the tool, leaving a straight step with a width of 8.4 mm. Four side lengths are analyzed: 25 mm, 12.5 mm, 10 mm and 8 mm. Again, except for the 25 mm side length (SL), two steps are milled to leave also a straight shoulder.

**Figure 5:**
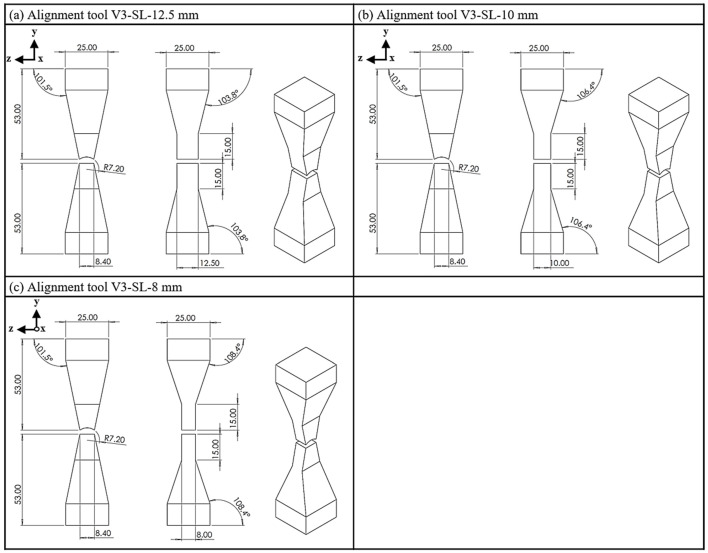
Tool V3; (**a**) side length, 12.5 mm; (**b**) side length, 10 mm; (**c**) side length, 8 mm.

Alignment tool V3 represents an optimized version of tools V1 and V2, characterized by a conical area in the middle section of the yokes and a straight shoulder in the z-direction in the area of the strand, as seen in tool V2 (Figure 5). The testing process involves utilizing a shoulder with side lengths of 12.5 mm, 10 mm and 8 mm.

To gain a more profound understanding of the trajectory of the field lines, a magnetic field simulation was conducted. FEMM (Finite Element Method Magnetics, version 4.2) software was used for simulation. This software allows for 2D simulation of magnetic problems. As it is a 2D simulation, the magnetic flux is only simulated from two planes, which introduces a systematic error because, in reality, the field lines also flow into the third plane. This systematic error is neglected in this work. For a more accurate simulation of the tool types, a 3D simulation program is required. The setup of the simulation and all simulation values can be found in Appendix B.

**Figure 6:**
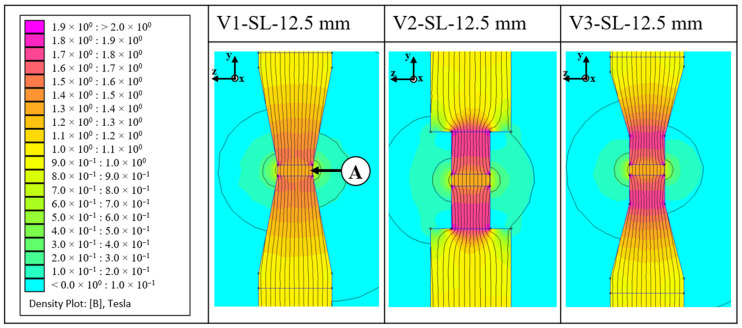
Magnetic flux simulation of the alignment tool types; A: NdFeB feedstock.

**Figure 7:**
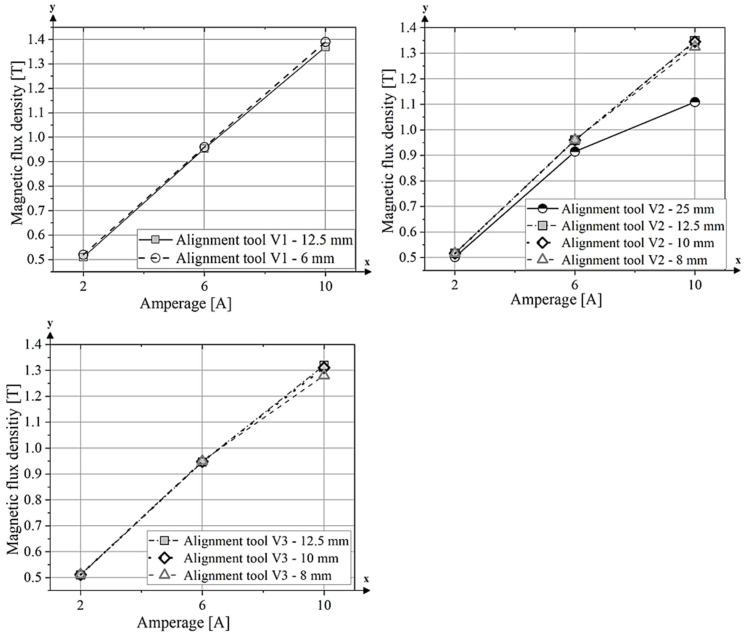
Results of the simulation of the magnetic flux density B, measured at the centre of the extruded part.

The simulation outcomes for the lateral perspectives of the three tool variants, each with a side length of 12.5 mm, are presented in Figure 6. The extruded part made from NdFeB feedstock is located between the upper and lower yokes at the measurement point. Simulation results for all alignment tools under different magnetic field conditions are shown in Figure 7. The measurement point is located at the centre of the extruded part.

Variant V1 shows a uniform increase in magnetic flux along the length of the yoke. Variant V2 exhibits an abrupt reduction in cross-section, leading to a sudden increase in magnetic flux density. Variant V3 follows the same pattern as variant V1, with field lines being evenly guided through the yoke. However, there is a greater increase in magnetic flux density due to the larger reduction in cross-section. The results, measured in the centre of the green part, show a clear increase in magnetic flux density as the induced current in the electromagnet increases. With an applied current of 10 A, a magnetic field of 1.4 T is achieved for variant V1 with a side length of 6 mm and 1.37 T for the tool with a side length of 12.5 mm. However, it is notable that these values are almost identical for both side lengths. The lowest value at 10 A induced current was achieved with variant V2–25 mm, with a value of 1.11 T. The values for tool 3 are lower overall compared to tool variants 1 and 2. With a side length of 12.5 mm and 10 A current, the value is 1.3 T.

### 2.4. Debinding and Sintering

In contrast to the process of producing polymer-bonded NdFeB permanent magnets, which does not necessitate the removal of the polymer, MIM/PEM necessitates the removal of the polymer and subsequent sintering of the green parts. Polymer-bonded NdFeB permanent magnets can be used directly after shaping and magnetization. However, this is not possible with MIM/PEM green parts due to the mechanical instability and low thermal stability of the green parts, and the magnetic properties are also very low due to the polymer in the structure. Subsequently, the green parts were debinded in a solvent solution following the shaping process with extrusion. To remove the main binder, the samples are placed in the solvent for a period of 24 h at a temperature of 60 °C, with continuous stirring. The samples are then placed in the sintering furnace, where the initial step is to remove the backbone binder. This is achieved by gradually increasing the temperature in hydrogen to 650 °C at a heating rate of 30 K/h, maintaining this temperature for 30 min and ensuring the complete removal of the binder. Once the thermal debinding process is complete, the gas atmosphere within the furnace is switched from hydrogen to argon. The debinded specimens are then sintered by heating the furnace to 1100 °C. The thermal debinding and sintering process was conducted using a hinged furnace FST 12/60/500 from Carbolite Gero GmbH & Co. KG (Neuhausen, Germany). As the Curie temperature is exceeded during the sintering process, it is necessary to magnetize the samples after sintering. This was achieved using a K-series pulse magnetiser from Magnet Physik Dr. Steingroever GmbH (Cologne, Germany), which was used to subject the samples to three pulses of 2000 V parallel to the easy axis.

Once the samples had been magnetized parallel to the easy axis, the magnetic properties were measured using a Hystograph HG 200 from Dr. Brockhaus Messtechnik GmbH & Co. KG (Lüdenscheid, Germany) at room temperature. The magnetized samples were measured parallel to the alignment axis (easy axis) and perpendicular to the easy axis (hard axis). The measurement of all samples was carried out using a measuring coil with an opening of 10 mm diameter. To ensure the reliability of the hysteresis measurements, repeated measurements were performed using the sintered NdFeB starting magnet (prior to hydrogen treatment) as an internal reference. The calculated standard deviations based on multiple measurements were 0.27% for remanence *B_r_*, 0.30% for coercivity *H_cJ_*, 0.28% for saturation polarization *J_s_* and 0.51% for the maximum energy product BH_max_. The maximum uncertainty of the *B_r_*/*J_s_* ratio was conservatively estimated using Gaussian error propagation, assuming a correlation coefficient of R = 0.95. This results in a combined standard deviation of ±0.0038 (±0.38%), based on the maximum relative uncertainties of *B_r_* = (0.27%) and *J_s_* = (0.28%).

The maximum energy product *BH*ₘₐₓ is a key figure of merit for quantifying the performance of permanent magnets. It indicates the maximum magnetic energy that can be stored per unit volume and is determined from the second quadrant of the demagnetization curve. *BH*_max_ corresponds to the point at which the product of magnetic flux density *(B)* and magnetic field strength *(H)* reaches its maximum, geometrically forming the largest rectangle under the *B–H* curve. In this study, *BH*_max_ was automatically calculated during hysteresis measurements using the Hystograph HG 200 system (Dr. Brockhaus Messtechnik GmbH & Co. KG, Lüdenscheid, Germany). All deviations remained below 1%, confirming the reproducibility of the magnetic characterization. The micrographs were captured utilizing a FlexSEM 1000 scanning electron microscope (Hitachi High-Tech GmbH, Krefeld, Germany). The density of the samples was determined by using the Archimedes method with isopropanol as the immersion liquid.

## 3. Results and Discussion

### 3.1. Characterization of the Starting Material for the Extrusion Process

The starting material for these experiments was obtained from an end-of-life (Eol) wind turbine magnet. The magnetic properties of the sintered starting magnet and those of an isotropic PEM magnet produced from the same recycled material are compared (Table 2).

**Figure A1:**
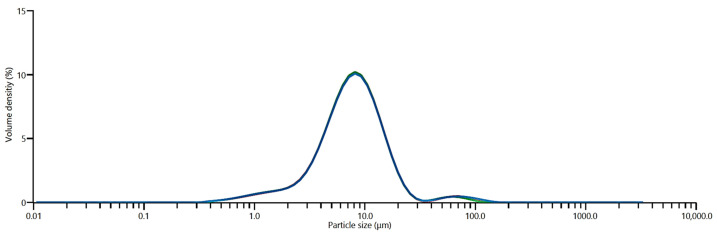
Particle size distribution of the milled powder measured using a Mastersizer 3000 (Malvern Panalytical GmbH, Nuremberg, Germany). The different colors represent three independent measurements of the same powder sample.

The particle size distribution after milling was characterized by a median size D_(50)_ of 7.68 µm, with D_(10)_ and D_(90)_ values of 2.97 µm and 16.1 µm, respectively. The full distribution curve is presented in Appendix A (Figure A1). As demonstrated by Sun et al. [26], the degree of magnetic alignment is strongly influenced by particle size. Their study showed that a particle size of approximately 4.9 µm yields the highest degree of alignment. Larger particles as those used in the present study possess a polycrystalline structure characterized by multiple crystal grains with distinct crystallographic orientations. Each of these crystal grains exhibits a unique anisotropy easy axis. Due to the variation in easy axes among the grains, the external magnetic field cannot achieve optimal alignment across all grains. Consequently, some grains exhibit only partial alignment with the applied field, while others retain an unfavourable magnetization direction.

### 3.2. Extrusion with Alignment Tool V1

**Figure 8:**
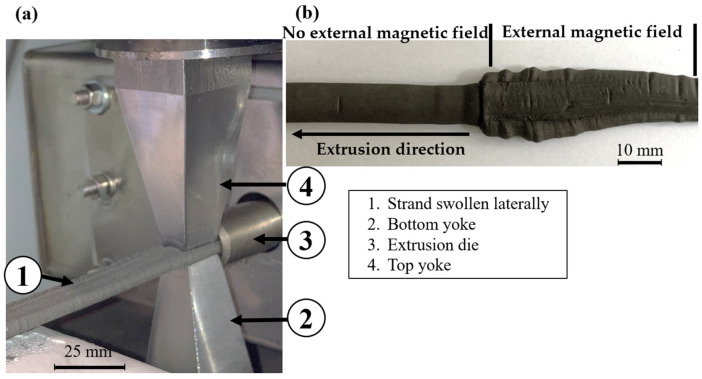
(**a**) Alignment tool V1 and a laterally swollen strand; (**b**) a laterally swollen strand.

The V1–12.5 mm alignment tool is illustrated in Figure 8a. The strand is formed in the extrusion die (3), and the magnetic particles are then aligned by the lower yoke (2) and upper yoke (4). After the strand leaves the die and passes through the magnetic field, lateral swelling of the strand occurs, (Figure 8b). This effect occurs with both 12.5 mm and 6 mm tool lengths as soon as the magnetic field is switched on. Even at low magnetic field strengths, generated by an induced current of 2 A, the strand swells. Based on the simulation results, a magnetic field of 0.52 T, generated by a current of 2 A in the electromagnet, would be sufficient to prevent a proper extrusion process.

**Figure 9:**
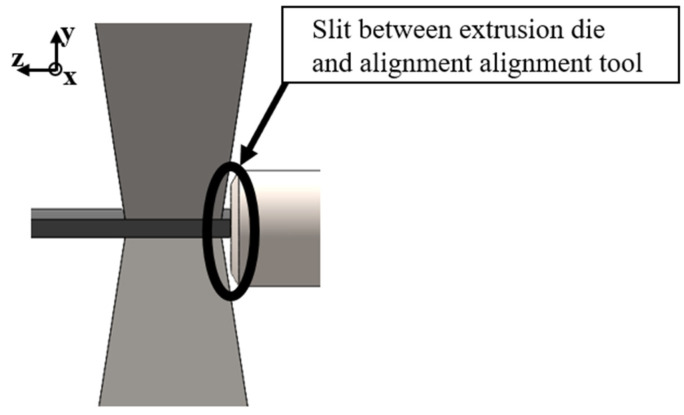
Slit between extrusion die and alignment tool V1.

The cause of this effect is unclear, but as it only occurs with tool variant 1, i.e., the variant where both yokes are conical in the extrusion direction, it can be assumed that it is related to this design. A possible explanation of the problem is shown in Figure 9.

The conical shape of the alignment tool creates a slit between the extrusion die and the alignment tool. As a consequence, the strand can be attracted by the bevels of the conical yoke when the magnetic field is switched on, thus impeding the constant flow. As the strand adheres to the tool, the material is unable to continue to flow and therefore deviates to the side.

As it was not possible to produce a suitable strand with this type of tool, no further measurements or analyses were carried out.

### 3.3. Extrusion with Alignment Tools V2 and V3

**Figure 10:**
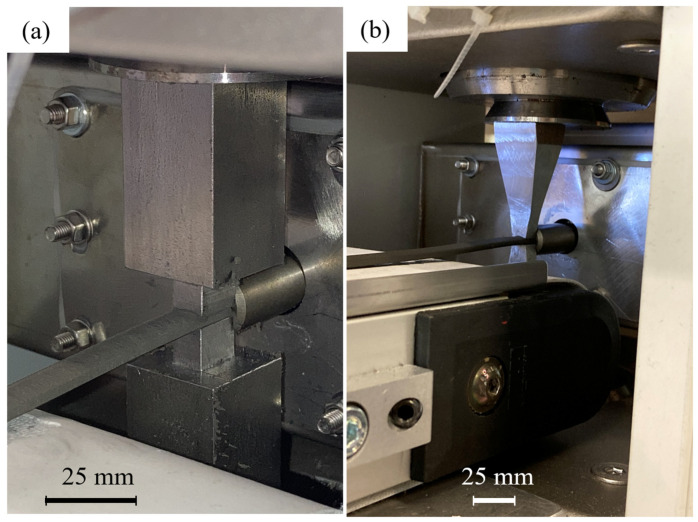
Extrusion process with alignment tool; (**a**) alignment tool V2–12.5 mm; (**b**) alignment tool.

In the case of alignment tools V2 and V3, whose sides are not conical but straight in the extrusion direction, the strand was able to flow through all the applied magnetic fields without encountering any problems, as illustrated in Figure 10.

**Figure 11:**
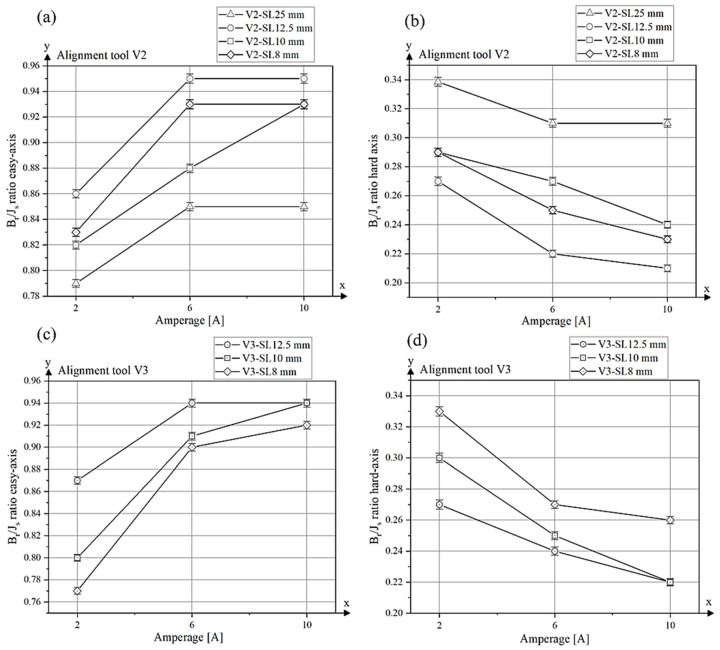
*B_r_/J_s_* ratio along the easy axis and hard axis of alignment tools V2 (**a**,**b**) and V3 (**c**,**d**) under different magnetic field strengths.

The ratio *B_r_/J_s_* along the easy magnetization axis is plotted as a function of current (amps) for different tool sizes (25 mm, 12.5 mm, 10 mm and 8 mm) of the alignment tool V2 (Figure 11a). For all tool lengths, the *B_r_/J_s_* ratio increases significantly from 2 A to 6 A, indicating improved magnetic alignment with increasing field strength. However, between 6 A and 10 A, the values for the V2 tools with side lengths of 12.5 mm, 8 mm and 25 mm reach a plateau, indicating that a further increase in field strength does not significantly improve particle alignment. In contrast, the V2–10 mm tool shows a continuous increase in the *B_r_/J_s_* ratio from 6 A to 10 A. Since the *B_r_/J_s_* values for the V2-SL–10 mm tool are lower than those of the V2-SL–12.5 mm and V2-SL–8 mm tools at 6 A, it is likely that the magnetic alignment was suboptimal at this current. One possible explanation for this is a slight displacement of the die during extrusion, which may have prevented the magnetic field from being properly focused on the extruded strand.

**Figure 12:**
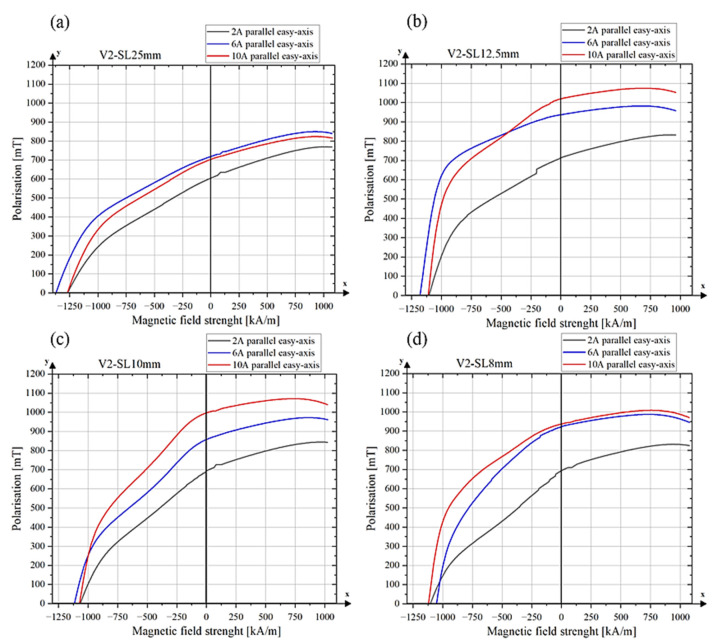
Demagnetization curves of sintered samples (**a**) alignment tool V2-SL–25 mm, (**b**) alignment tool V2-SL–12.5 mm, (**c**) alignment tool V2-SL–10 mm, (**d**) alignment tool V2–8 mm.

The magnetic properties of all sintered NdFeB samples produced using alignment tool V2 are summarized in Table 3, and the corresponding demagnetization curves are presented in Figure 12. The highest degree of alignment along the easy axis was obtained using the V2 alignment tool with a side length of 12.5 mm at field strengths generated by currents of 6 A and 10 A, resulting in a *B_r_/J_s_* ratio of 0.95. The configuration V2-SL–12.5 mm-10 A also yielded the highest remanence (*B_r_* = 1019.66 mT) and saturation polarization (*J_s_* = 1074.49 mT) among all tested samples as well as the highest maximum energy product (*BH*_max_ = 160.97 kJ/m^3^).

Sample V2-SL–10 mm-10 A also exhibited high saturation values (*J_s_* = 1071.61 mT) and a remanence *B_r_* of 996.83 mT, resulting in a slightly lower *B_r_/J_s_* ratio of 0.93.

In contrast, the lowest degree of alignment was observed for sample V2-SL–25 mm-2 A, which was processed with a low alignment field. This sample showed a *B_r_/J_s_* ratio of only 0.79, a remanence of 604.74 mT and a saturation polarization of 769.83 mT. These values are comparable to those typically observed in isotropic NdFeB magnets produced by the powder extrusion process [12]. In contrast to the measurement parallel to the alignment axis, the *B_r_/J_s_* ratio parallel to the hard axis is shown (Figure 11b). Here, the values decrease with increasing magnetic field and decreasing tool surface, as the magnetic moments are better aligned in the easy axis. The tool with a side length of 25 mm exhibited the highest *B_r_/J_s_* value of approximately 0.34 at a current of 2 A, demonstrating only a marginal decline throughout the measurement process. It attained a value of 0.31 at 10 A. The remaining tool sizes (12.5 mm, 10 mm and 8 mm) demonstrate a substantially more pronounced decline in the *B_r_/J_s_* ratio at elevated currents. The tool with a side length of 12.5 mm has an initial value of approximately 0.27 at 2 A, which decreases to 0.21 at 10 A. Similar behaviour can be observed for the tools with 10 mm and 8 mm side length, as the values here also decrease with stronger magnetic fields.

The results for the degree of alignment parallel to the easy axis for tool type V3 are shown in Figure 11c, and the corresponding values along the hard axis are presented inFigure 11d. The results exhibit similar trends to those observed for tool type V2. An increase in the *B_r_/J_s_* ratio is observed between 2 A and 6 A for all tool lengths, indicating enhanced magnetic particle alignment along the easy axis. For samples V3-SL–10 mm and V3-SL–8 mm, a further increase in the *B_r_/J_s_* ratio is observed between 6 A and 10 A, whereas for V3-SL–12.5 mm, the values remain constant at higher fields. The alignment tool V3-SL–12.5 mm achieves the highest degree of alignment with a *B_r_/J_s_* ratio of 0.94 at both 6 A and 10 A. Sample V3-SL–10 mm also reaches a value of 0.94 at 10 A. Furthermore, tool V3-SL–12.5 mm exhibits the highest remanence (*B_r_* = 986.81 mT) and saturation polarization (*J_s_* = 1048.38 mT) among all V3 samples. The magnetic values of tool variant V3 are shown in Table 4 and the demagnetization curves in Appendix C.

Compared to isotropic NdFeB magnets processed via PEM in previous experiments [12], which exhibit a *B_r_/J_s_* ratio of approximately 0.78, the optimized anisotropic samples in this study reach values up to 0.95. This indicates a significantly improved magnetic alignment achieved during extrusion applying suitable tool geometry and magnetic field strength.

The demagnetization curves of V2-SL–12.5 mm-6 A and 10 A display a more pronounced squareness than that of the 2 A sample (Figure 12a). However, the curves still show signs of a gradual and incoherent reversal of magnetic domains, which limits the squareness and prevents the curves from reaching an ideal rectangular shape. Similar curve shapes can be seen for V2-SL–10 mm, except that the coercivity is slightly lower (Figure 11c). A lower coercivity leads to an earlier and more gradual decline in the second quadrant of the hysteresis curve, resulting in reduced squareness.

This reduced coercivity leads to an earlier and more gradual decline in the second quadrant of the hysteresis curve, resulting in a lower degree of squareness. Such behaviour is consistent with descriptions in the literature [27], where similar kinks have been attributed to the presence of grains with reduced coercivity, often located near the surface. These grains undergo magnetization reversal at lower opposing fields, leading to a noticeable change in the slope of the curve. This deviation is typically associated with microstructural defects or insufficient decoupling of the grains.

The demagnetization curves of the V2-SL–25 mm tool variant show low squareness in the second quadrant. While the squareness improves slightly with increasing magnetizing field, it remains suboptimal.

The low squareness for the V2–25 mm sample can be attributed to a limited degree of magnetic alignment. According to the Stoner–Wohlfarth model [28], which describes the magnetization reversal of single-domain particles with uniaxial anisotropy, a rectangular demagnetization curve occurs when the particle’s easy axis is fully aligned with the external field (0° misalignment). This ideal case assumes uniform rotation, no demagnetizing effects and zero temperature. If the magnetic field is misaligned (e.g., 10°), the hysteresis loop deviates from an ideal rectangular shape, reflecting a more progressive magnetization reversal dominated by anisotropy energy.

**Figure 13:**
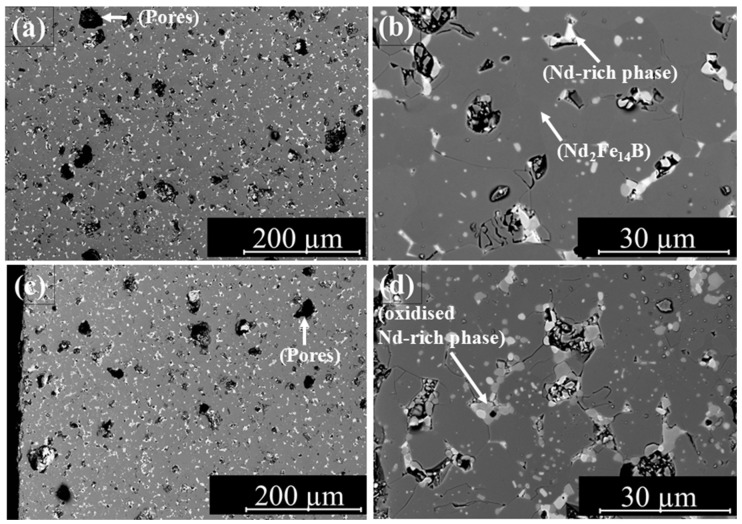
Microstructure of sample V2–12.5 mm-10 A; middle zone (**a**,**b**); left edge (**c**,**d**).

The microstructure of sample V2–12.5-10 A, which exhibited the best magnetic properties, is shown in Figure 13. Figure 13a,b show the middle region of the sample cross-section at different magnifications, and Figure 13c,d show the left edge at various magnifications. The micrographs reveal the presence of numerous pores in the sintered samples. These pores act as defects, impeding the process of nucleation and, consequently, hindering the reversal of magnetization.

The micrographs reveal a fine-grained structure with a well-distributed Nd-rich phase throughout the area. However, pores of different sizes can be seen within the microstructure. Larger pores may result from gas inclusions, as vapours generated during thermal debinding can become trapped within the part. Additionally, oxidized NdH_2.7_ may detach from the microstructure during sample preparation, contributing to porosity.

Pores and structural defects in the microstructure affect magnetization reversal by serving as preferred nucleation sites for reversed magnetic domains. As a result, demagnetization initiates locally at lower opposing fields, leading to a reduced coercive field. Additionally, such defects can act as pinning centres that impede the propagation of Domain walls. This results in a stepwise magnetization reversal and decreases the squareness ratio.

The coercivity of the measured samples ranges from 1000 kA/m to 1300 kA/m. In addition to microstructural defects such as pores, variations in particle size distribution as well as the shape and size of the magnet can also influence the coercivity. However, these effects can be considered negligible in the case of the observed differences in coercive field strength in this study, as all samples were produced from the same powder batch and had identical dimensions. In addition, geometric influences were compensated by the standard software provided with the Hystograph HG 200 (Dr. Brockhaus Messtechnik GmbH & Co. KG, Lüdenscheid, Germany), which corrects the demagnetizing fields based on the entered sample dimensions and weight. The observed variations in coercivity are therefore most likely due to pores and oxidation effects at the edge zones of the samples that occur during sintering. In particular, the Nd-rich grain boundary phase is prone to oxidation, especially in the edge regions of the samples, if the furnace atmosphere is not sufficiently controlled. Once oxidized, the Nd-rich phase loses its ability to effectively wet and separate the magnetic grains, leading to magnetic coupling and, consequently, a reduction in coercivity.

Although a relative density of approx. 96% was achieved in this study, the rema-nence *B_r_* and saturation polarization *J_s_* remain below those of the starting magnet. This can be explained by residual porosity and possible microstructural changes, such as partial oxidation or incomplete grain alignment. These factors reduce the effective fraction of the magnetically active phase. However, the primary reason for the reduced *B_r_* and *J_s_* in this case is likely the incomplete alignment of the magnetic particles during processing.

If the Nd-rich intergranular phase does not fully separate the grains, magnetic coupling can occur via exchange interactions. These coupled grains tend to reverse their magnetization collectively, which reduces the coercivity of the material. Micromagnetic simulations have shown that such exchange coupling can decrease the coercive field to approximately 40% of the ideal nucleation field [29].

## 4. Summary/Further Work

The aim of this work was to prove that in situ, anisotropic NdFeB permanent magnets can be produced using the PEM process. To provide this proof, alignment tools were designed that can concentrate the magnetic field on the strand. Extrusion tests were carried out with these tools at various magnetic field strengths. It was found that the magnets with the highest degree of alignment were produced using a tool with an edge length of 12.5 mm and induced current of 10 A applied to the electromagnet. It has also been shown that magnetic field strength has an influence on the orientation of the magnetic particles during the PEM process. The samples with a magnetic field generated at 2 A are almost isotropic for all tool variants. Increasing the current to 6 A and 10 A shows a significant improvement in the degree of alignment. The geometry of the yokes also has an influence, particularly the reduction in the edge length from 25 mm to 12.5 mm had an enormous influence on the alignment behaviour, as it made it possible to increase the applied magnetic field.

The highest degree of magnetic alignment was achieved using the tool with a side length of 12.5 mm and an applied magnetic field generated by a current of 10 A, resulting in a *B_r_/J_s_* ratio of 0.95. The shape of the demagnetization curve is dependent on the alignment of the particles and the quality of the microstructure; both the alignment of the magnetic particles and the microstructure still need to be optimized. The pores present in the microstructure serve as pinning centers, impeding the movement of domain walls and resulting in a stepwise magnetization reversal instead of an abrupt change.

Optimization of the alignment of the extruded green parts can be achieved by using finer powder. In future studies, experiments with different particle sizes should be carried out to determine the optimum particle size for the alignment of magnetic particles with PEM. The microstructures show pores of different sizes, which have a negative effect on the density and the saturation *J_s_* of the sample. The thermal debinding and sintering processes need to be optimized in a further development step. In addition, tests with jet-milled powder also need to be carried out in order to produce denser magnets.

It is imperative to conduct additional experimentation with varying extrusion temperatures, given the substantial impact of the binder’s viscosity on the orientation of the magnetic particles.

## Figures and Tables

**Table 1:** Extrusion parameters.

Extrusion Parameters	Values
Temperature Zone 1 [°C]	80
Temperature Zone 2 [°C]	170
Temperature Zone 3 [°C]	175
Temperature Zone 4 [°C]	175
Temperature Zone 5 [°C]	175
Temperature Zone 6 [°C]	180
Screw speed slow [rpm]	7
Haul-off speed slow [m/min]	0.1
Powder loading [%]	50
Amperage 1 [A]	2
Amperage 2 [A]	6
Amperage 3 [A]	10

**Table 2:** Magnetic values of Eol magnet and isotropic PEM magnet. The data on isotropic PEM magnets were obtained from [12].

Sample	*B_r_*∥[mT]	*J_s_*∥[mT]	$\frac{B_{r}}{J_{s}}Ratio$	Density [g/cm^3^]
Eol magnet	1194	1247	0.96	7.50
PEM magnet, isotropic	531	678	0.78	7.28

**Table 3:** Magnetic values and density of samples aligned with alignment tool V2.

Sample	Coercivity [kA/m]	Remanence [mT]	Saturation [mT]	*BH*_max_ [kJ/m^3^]	*B_r_/J_s_* Ratio ‖Easy Axis	*B_r_/J_s_* Ratio ‖Hard Axis	Density [g/cm^3^]
V2-SL–25 mm-2 A	1269.20	604.74	769.83	59.12	0.79	0.34	7.32
V2-SL–25 mm-6 A	1372.37	718.11	849.68	86.40	0.85	0.30	7.31
V2-SL–25 mm-10 A	1271.61	703.24	823.93	80.88	0.85	0.29	7.29
V2-SL–12.5 mm-2 A	1103.79	713.76	832.85	80.99	0.86	0.27	7.30
V2-SL–12.5 mm-6 A	1179.55	936.95	982.36	152.24	0.95	0.22	7.28
V2-SL–12.5 mm-10 A	1110.50	1019.66	1074.49	160.97	0.95	0.21	7.27
V2-SL–10 mm-2 A	1065.13	693.92	844.27	69.97	0.82	0.27	7.30
V2-SL–10 mm-6 A	1114.84	857.75	972.89	105.71	0.88	0.24	7.20
V2-SL–10 mm-10 A	1072.21	996.83	1071.61	142.27	0.93	0.23	7.26
V2-SL–8 mm-2 A	1103.98	693.47	831.72	68.74	0.83	0.31	7.31
V2-SL–8 mm-6 A	1052.09	922.96	987.40	132.62	0.93	0.29	7.23
V2-SL–8 mm-10 A	1119.89	937.38	1008.87	142.65	0.93	0.25	7.24

**Table 4:** Magnetic values and density of samples aligned with alignment tool V3.

Sample	Coercivity [kA/m]	Remanence [mT]	Saturation [mT]	*BH*_max_ [kJ/m^3^]	*B_r_/J_s_* Ratio ‖Easy-Axis	*B_r_/J_s_* Ratio ‖Hard-Axis	Density [g/cm^3^]
V3-SL–12.5 mm-2 A	1145.61	691.67	798.49	73.83	0.87	0.27	7.30
V3-SL–12.5 mm-6 A	1279.49	947.34	1002.84	148.79	0.94	0.24	7.29
V3-SL–12.5 mm-10 A	1190.18	986.81	1048.38	138.19	0.94	0.22	7.27
V3-SL–10 mm-2 A	1273.35	549.31	686.53	51.71	0.80	0.30	7.31
V3-SL–10 mm-6 A	1120.33	898.43	982.04	129.17	0.91	0.25	7.22
V3-SL–10 mm-10 A	1119.63	953.25	1017.71	142.56	0.94	0.22	7.29
V3-SL–8 mm-2 A	1005.81	549.69	711.42	47.72	0.77	0.33	7.29
V3-SL–8 mm-6 A	1139.50	740.43	822.37	92.98	0.90	0.27	7.22
V3-SL–8 mm-10 A	1058.42	897.55	977.53	122.58	0.92	0.26	7.19

**Figure A2:**
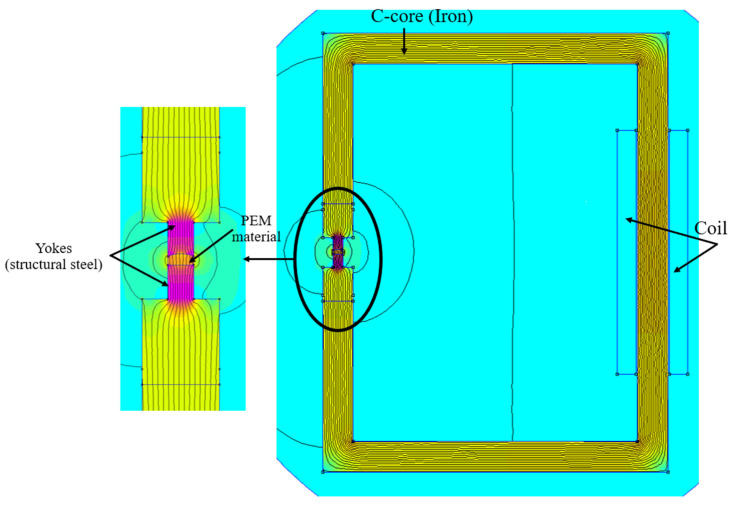
Simulation setup.

**Figure A3:**
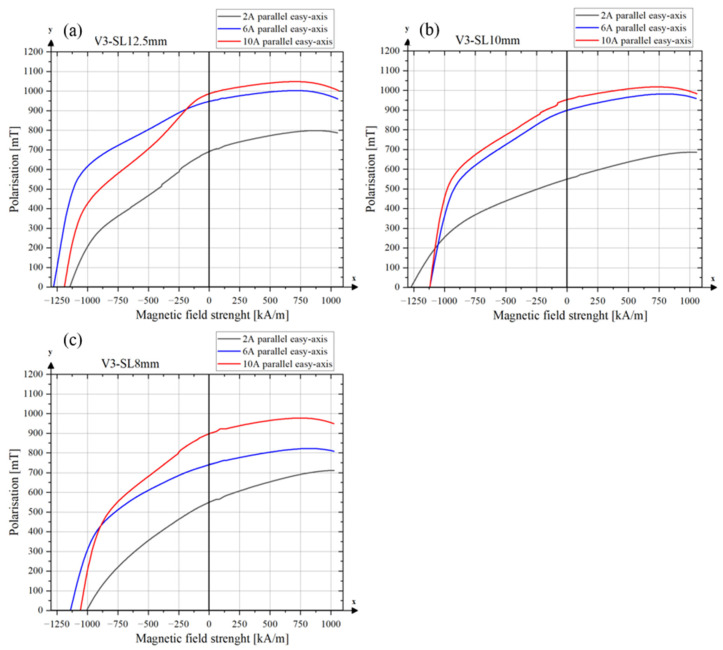
Demagnetization curves of sintered samples. (**a**) Alignment tool V3-SL–12.5 mm; (**b**) alignment tool V3-SL–10 mm; (**c**) alignment tool V3-SL–8 mm.

## Data Availability

The data presented in this study are available on request from the corresponding authors.

## Abbreviations

*BH*_max_	Maximum energy product
*B_r_*	Remanence
EBSD	Electron Backscatter Diffraction
Eol	End-of-life
EU	European Union
FFF	Fused Filament Fabrication
FEM	Finite Element Method
FEMM	Finite Element Method Magnetics
HPMS	Hydrogen Processing of Magnetic Scrap
*H_cJ_*	Coercivity
*J_s_*	Saturation polarization
MEX	Material Extrusion
MIM	Metal Injection Molding
NdFeB	Neodymium-Iron-Boron
PA6/PA12	Polyamide 6/Polyamide 12
PE	Polyethylene
PEM	Powder Extrusion Molding
REE	Rare Earth Elements
REPM	Rare Earth Permanent Magnets
VSM	Vibrating Sample Magnetometer
XRD	X-ray Diffraction

## Appendix B

**Table A1:** Values simulation.

Windings	Values
Relative permeability µ_r_	1
Electrical conductivity [MS/m]	58
Quantity	300
Diameter [mm]	1
C-Core	
Relative permeability µ_r_	14,872
Electrical conductivity [MS/m]	10.44
Yoke structural steel	
Relative permeability µ_r_	760
Electrical conductivity [MS/m]	5.8
PEM-material	
Relative permeability µ_r_	1.1
Electrical conductivity [MS/m]	0.01
